# Risk factors of instrumentation failure after laminectomy and posterior cervical fusions (PCF)

**DOI:** 10.1186/s12891-023-07116-z

**Published:** 2024-01-02

**Authors:** Zejun Chen, Guohua Lv, Ou Zhang, Yunchao Li, Xiaoxiao Wang, Haoyu He, Hui Yuan, Changyu Pan, Lei Kuang

**Affiliations:** 1 Department of Spinal Surgery, The Second Xiangya Hospital of Central South University, Changsha, Hunan Province China; 2 California University of Science and Medicine, Colton, CA USA

**Keywords:** Hardware failure, Laminectomy and posterior cervical fusion, Degenerative cervical myelopathy, Osteoporosis, Hounsfield units, Screw density, Sagittal alignment

## Abstract

**Background:**

For patients with multilevel degenerative cervical myelopathy, laminectomy and posterior cervical fusions (PCF) with instrumentation are widely accepted techniques for symptom relief. However, hardware failure is not rare and results in neck pain or even permanent neurological lesions. There are no in-depth studies of hardware-related complications following laminectomy and PCF with instrumentation.

**Methods:**

The present study was a retrospective, single centre, observational study. Patients who underwent laminectomy and PCF with instrumentation in a single institution between January 2019 and January 2021 were included. Patients were divided into hardware failure and no hardware failure group according to whether there was a hardware failure. Data, including sex, age, screw density, end vertebra (C7 or T1), cervical sagittal alignment parameters (C2-C7 cervical lordosis, C2-C7 sagittal vertical axis, T1 slope, Cervical lordosis correction), regional Hounsfield units (HU) of the screw trajectory and osteoporosis status, were collected and compared between the two groups.

**Results:**

We analysed the clinical data of 56 patients in total. The mean overall follow-up duration was 20.6 months (range, 12–30 months). Patients were divided into the hardware failure group (n = 14) and no hardware failure group (n = 42). There were no significant differences in the general information (age, sex, follow-up period) of patients between the two groups. The differences in fusion rate, fixation levels, and screw density between the two groups were not statistically significant (p > 0.05). The failure rate of fixation ending at T1 was lower than that at C7 (9% vs. 36.3%) (p = 0.019). The regional HU values of the pedicle screw (PS) and lateral mass screw (LMS) in the failure group were lower than those in the no failure group (PS: 267 ± 45 vs. 368 ± 43, p = 0.001; LMS: 308 ± 53 vs. 412 ± 41, p = 0.001). The sagittal alignment parameters did not show significant differences between the two groups before surgery or at the final follow-up (p > 0.05). The hardware failure rate in patients without osteoporosis was lower than that in patients with osteoporosis (14.3% vs. 57.1%) (p = 0.001).

**Conclusions:**

Osteoporosis, fixation ending at C7, and low regional HU value of the screw trajectory were the independent risk factors of hardware failure after laminectomy and PCF. Future studies should illuminate if preventive measures targeting these factors can help reduce hardware failure and identified more risk factors, and perform long-term follow-up.

**Supplementary Information:**

The online version contains supplementary material available at 10.1186/s12891-023-07116-z.

## Background

Due to the rapid changes in modern production and lifestyle, the prevalence of cervical myelopathy is 3.8–17.6% [1]. Though the prevalence in various regions vary, the number of patients increases by year [1]. PCF with instrumentation is performed to treat degenerative diseases such as cervical myelopathy, ossification of the posterior longitudinal ligament (OPLL), and multilevel cervical radiculopathy [2]. Decompression relieves pressure on the spinal cord, and fixation helps correct and maintain cervical alignment and stability. Although there are various types of screws and techniques for screw insertion in the cervical spine, hardware failure is one of the most common complications [3–8]. Hardware failure is defined as screw or rod breakage, screw loosening, or nonunion. The failure rates ranged from 6.1 to 38.9% and may even exceed 50% after PCF [6, 7]. The incidence of hardware failure leading to surgical revision ranged from 16.7 to 42.8%, with a pooled incidence of 22.7% [2–6]. In addition, hardware failure may also cause pseudarthrosis, chronic pain, and neurologic deficits [2, 3, 6]. However, there have been few reports focusing on the characteristics and risk factors of hardware failure in laminectomy and PCF [3–5]. To assess this common postoperative complication, a thorough understanding of the characteristics and risk factors of hardware failure after laminectomy and PCF is needed. Therefore, we conducted the present study to elucidate the characteristics and risk factors of hardware failure in laminectomy and PCF.

## Patients and methods

### Study design

The present study was a retrospective, single centre, observational study. Data of patients following laminectomy and PCF with instrumentation between January 2019 and January 2021, including sex, age, screw density, end vertebra (C7 or T1), cervical sagittal alignment parameters (C2-C7 cervical lordosis (CL), C2-C7 sagittal vertical axis, T1 slope, CL correction), regional Hounsfield units (HU) of screw trajectory, and osteoporosis status were collected, which aimed to investigate risk factors of hardware failure after laminectomy and PCF with instrumentation. This study was approved by The Ethics Committee of The Second Xiangya Hospital of Central South University (NO.20,191,243) in January 2019. Written informed consent to participate in the study was obtained from each patients. This study is reported following the STROBE guidelines.

### Patient population

**Fig. 1:**
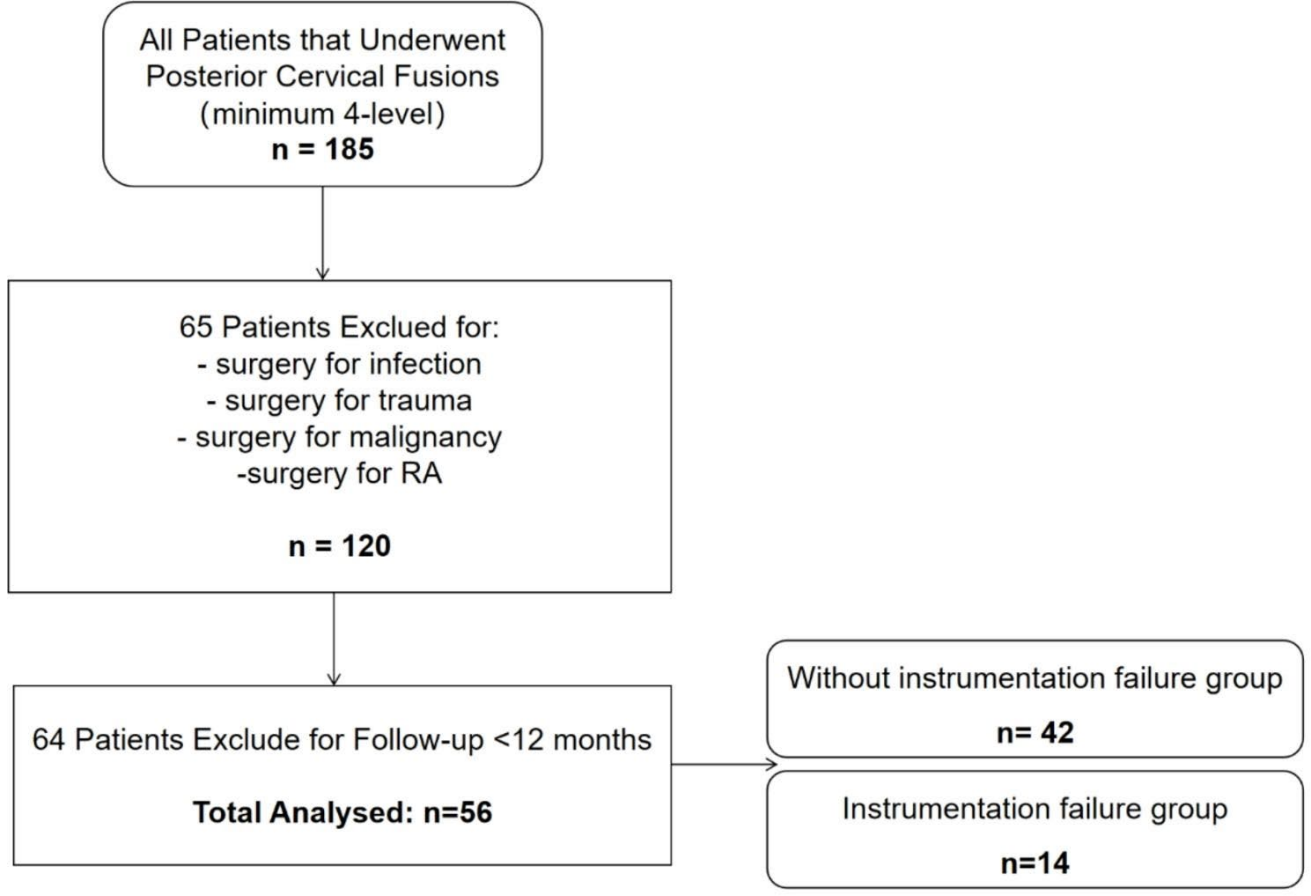
Flow chart showing patients selection

We accessed the inpatient information in the electronic medical records system of our hospital. The inclusion criteria were as follows: (1) patients who underwent 4-level and above laminectomy and PCF. The exclusion criteria were as follows: (1) follow-up less than 1 year; (2) age less than 18 years; and (3) cervical spinal surgery for infection, trauma, malignancy, or rheumatoid arthritis (RA). Patients were divided into hardware failure (n = 14) and no hardware failure group (n = 42) according to whether there was a hardware failure (Fig. 1).

### Surgical procedures

All patients underwent minimum 4-level laminectomy and PCF, and surgeries were performed by the same senior surgeon (Dr. Kuang). A midline incision was made followed by subperiosteal dissection of the paravertebral muscles to expose the spinous processes, laminae, and facet joints of the affected vertebrae. Lateral mass screws and pedicle screws of 3.5 or 4.0 mm diameter (Axon®, Synthes Inc., Raynham, MA, USA) were placed on the cervical vertebrae. Contoured rods of 3.5 mm diameter (Axon®, Synthes Inc., Raynham, MA, USA) were attached to the screws and locked with nuts. Radiographs were obtained to ensure accurate positioning of the screws and rods. Then, the laminae of the planned decompression segments were resected using a rongeur and high-speed bur. Prophylactic C4-5 foraminotomy was performed on patients with foraminal stenosis. Small wedges of autografts from the lamina were placed adjacent to bilateral joints to facilitate fusion.

### Outcome assessment

The fixation level, screw density (total number of screws/actual fixation level), regional HU of the PS/LMS screw trajectory, and status of osteoporosis were recorded. Osteoporosis was diagnosed by Dual energy X-ray absorptionmetry (DXA). T score is to compare the bone quality of the subject with that of a normal young man of the same sex to determine whether there is osteoporosis (Normal: -1 to + 1; Low bone mass: -1 to -2.5; Osteoporosis: less than − 2.5) [12].

Radiological examination was used to assess hardware failure. Osseointegrated screws do not show any sign of radiolucency around their edges in planar radiographs. Screw loosening was detected by the presence of a radiolucent area greater than 1 mm or the presence of a “double halo,” which is defined as an inner radiolucent zone surrounded by an outer radiopaque rim of dense bone [8]. Screw nut loosening occurred when the nut became dislodged from the screw head and could be seen as a gap between the screw grooves and the ridge [7]. Screw or rod breakage can be seen with obvious cracks and/or angulation in anteroposterior or lateral radiographs [8]. Nonunion was defined as a lack of bridging osseous trabeculae between the involved vertebrae [7], > 2 mm motion between the affected spinous processes on flexion-extension lateral radiographs, or > 2° of motion on flexion-extension radiographs at the 12-month follow-up [8]. When needed, computed tomography (CT) images were obtained to confirm the presence of nonunion. All patient imaging data (numbered but no patient information) were distributed to two spine surgeons (Dr. Pan and Dr. Yuan), who judged the occurrence of hardware failure based on the previous criteria, and if they agreed, no one else judged again. If there was a disagreement, a senior spine surgeon (Dr. Lv) was invited to participate in the judgement and make ultimate judgement.

**Fig. 2:**
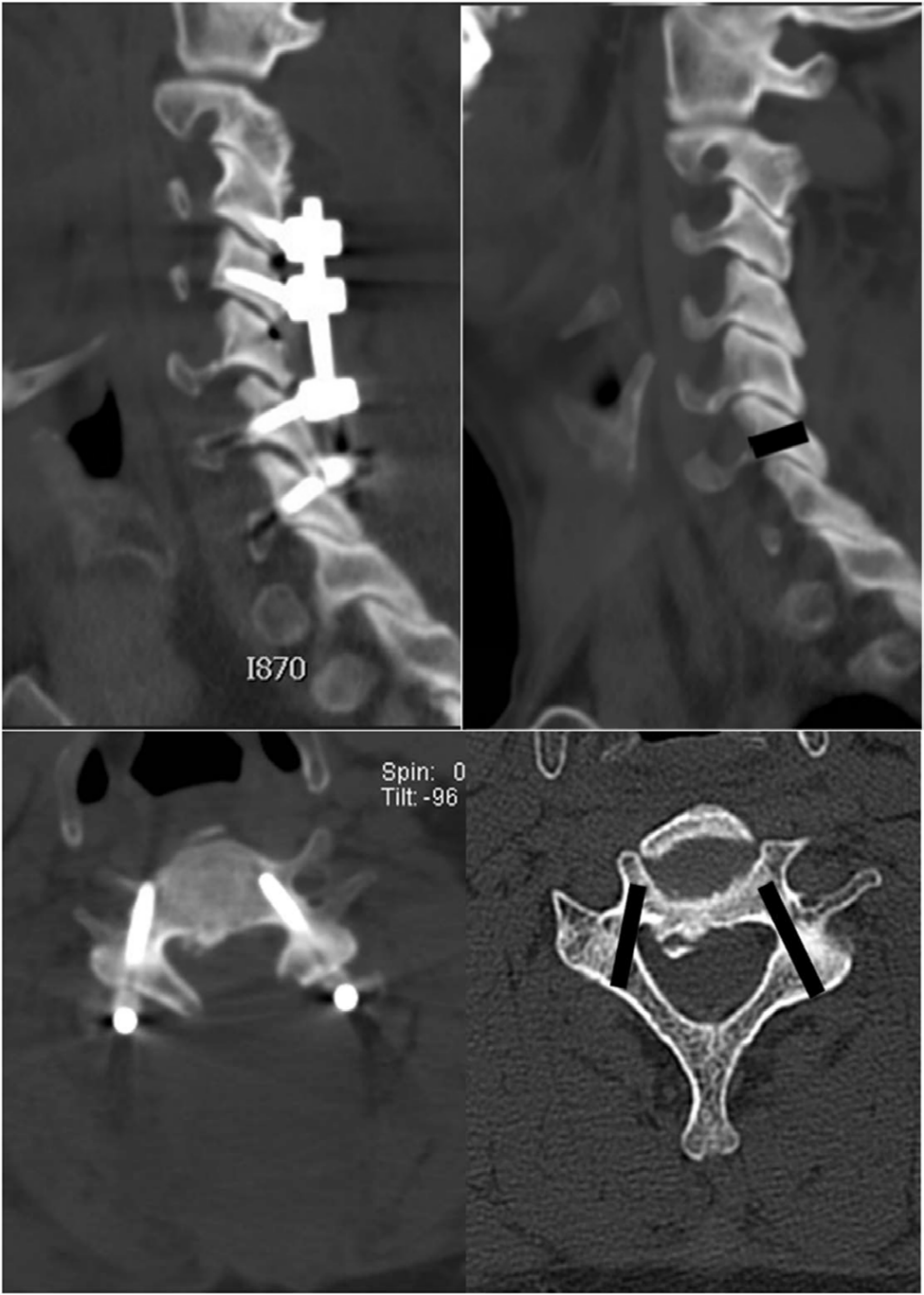
Extraction of cylindrical area of screw trajectory, screw position is confirmed by the postoperative CT

For HU measurement, all patients were assessed by a helical 64-channel CT scanner (Aquilion 64®, Toshiba Medical, Otawara, Japan). The position of each screw was extracted from postoperative CT images obtained immediately after surgery and superimposed three-dimensionally on the vertebra of the preoperative CT by referring to the vertebral anatomical landmarks (Fig. 2) [17]. The cylindrical area along each screw with an outer diameter was placed on the vertebra, and density information was collected for all voxels contacting the sample. Average HU values were calculated automatically from the entry point at the lamina to the screw tip per 1 mm section orthogonal to the screw axis.

**Fig. 3:**
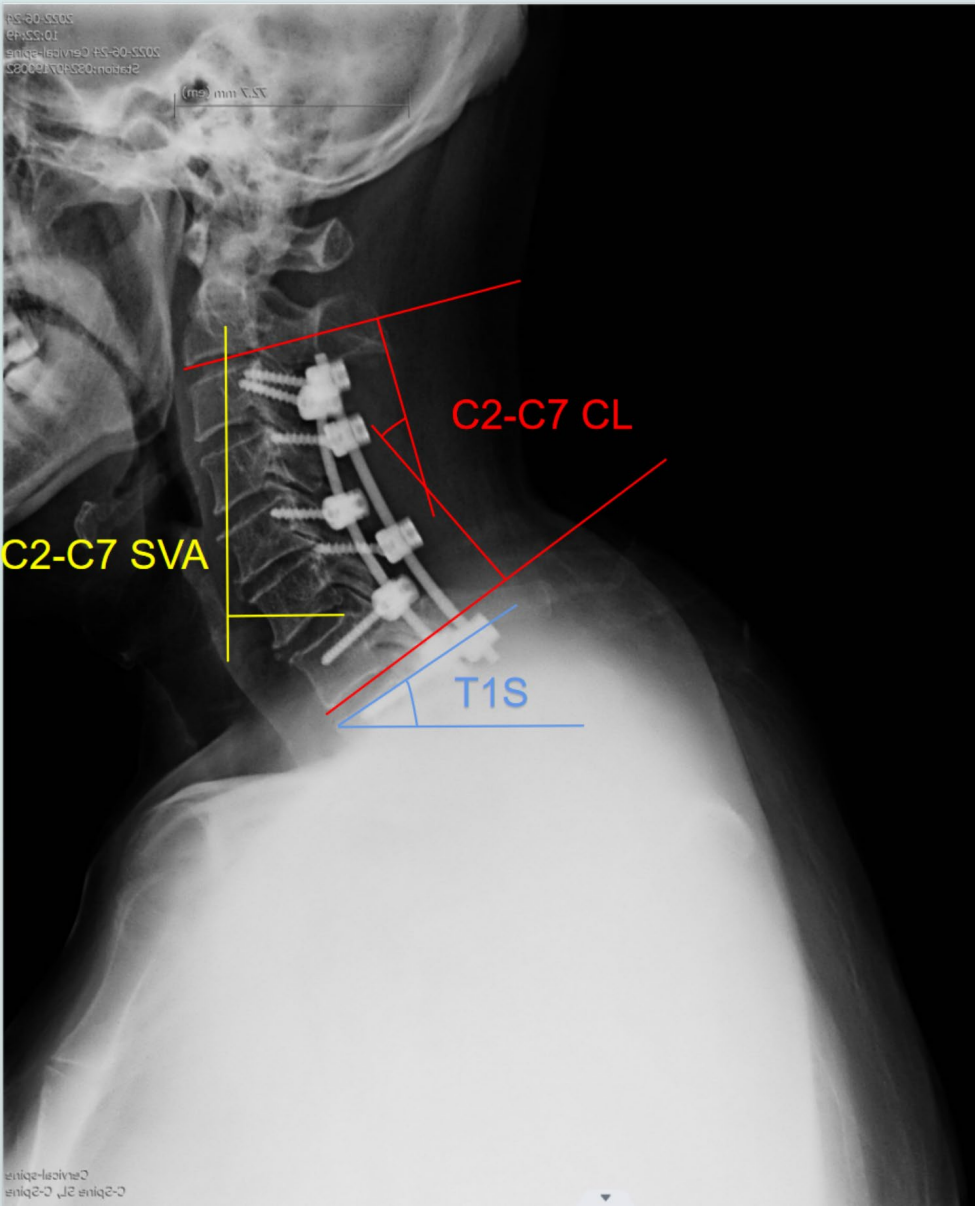
Radiological evaluation of the cervical sagittal alignment parameters. (1) C2-C7 sagittal vertical axis (SVA); (2) T1 slope (T1S); (3) C2-C7 cervical lordosis (CL). The C2-C7 SVA was obtained through measuring the distance from the posterior-superior corner of C7 to a vertical line that bisected the C2 centroid. The T1 slope is the angle created from a line tangential to the superior end plate of T1 and a horizontal line. Lastly, the C2-C7 CL was measured using the Cobb angle between the inferior end plate of C2 to the inferior end plate of C7

To evaluate the sagittal alignment, the following parameters through the cervical spine (standing position) radiograph were measured preoperatively and at the last follow-up: (1) C2-C7 sagittal vertical axis (C2-C7 SVA); (2) T1 slope (T1S); (3) C2-C7 Cervical lordosis (CL); (4) CL correction (postoperative C2-C7 CL minus preoperative C2-C7 CL) (Fig. 3).

All measurements were performed by two independent researchers (Dr. Pan and Dr. Yuan), and the results of their measurements were analysed by the intraclass correlation coefficient for data consistency. Disagreements were discussed with another independent expert (Dr. Lv), and a consensus was reached to minimize observer bias.

### Statistical analysis

The comparison between two groups was performed via Student’s t test, chi-square test and paired t tests in Statistical Product and Service Solutions 28.0 statistical software (SPSS, Inc., Chicago, Illinois, USA). The data are expressed as the mean and standard deviation (*x* ± S). The difference was statistically significant at *p* < 0.05. When the p-value was less than 0.001, it was recorded as 0.001.

## Results

### Clinical outcomes

We analysed the clinical data of 56 patients in total. The mean follow-up time was 20.6 months (range 12–30 months), and the average age of the patients was 55.6 years (range 36–81 years). Patients were divided into the hardware failure group (n = 14) and no hardware failure group (n = 42). There were no significant differences in the general information (age, sex, follow-up period) of the patients between the two groups. The differences in fusion rate, fixation level, and screw density between the two groups were not statistically significant (p > 0.05). We analysed a total of 479 screws (PS: 161, LMS:318), 30 of which had problems including screw loosening, breakage, or back out (PS:17, LMS:13). There was no loosening, breakage of the screw nut or rod breakage. The failure rate of the lower fixation endpoint at T1 was lower than that at C7 (9% vs. 36.3%, p = 0.019). The hardware failure rate in patients without osteoporosis was lower than that in patients with osteoporosis (14.3% vs. 57.1%, p = 0.001) (Table 1).

**Table 1:** General information and factors related to hardware failure

	Hardware failure	No hardware failure	*p*-value
No. of patients	14	42	
No. of screws	98 (30*)	381	
PS	37 (17*)	124	
LMS	61 (13*)	257	
Age (years)	57 ± 9.2	55 ± 9.6	0.515
Sex			0.086
Male/Female	10/4	31/11	
Fixation level	4.6 ± 0.7	4.5 ± 0.7	0.605
4 levels	7	26	
5 levels	5	10	
6 levels	2	6	
Fusion			0.987
With	13	39	
Without	1	3	
Screw density	1.5 ± 0.2	1.7 ± 0.3	0.051
Lower intrumented EV			0.019
C7	12	21	
T1	2	21	
Regional HU			
PS	267 ± 45	368 ± 43	0.001
LMS	308 ± 53	412 ± 41	0.001
Osteoporosis			0.001
With	8	6	
Without	6	36	

*Screws with hardware related problems including screws loosening, breakage, or back out

PS, pedicle screw; LMS, lateral mass screw; EV, end vertebra; Screw density (Number of screws divided by actually fixed levels)

### Radiological parameters

No patient in either group had any obvious instability or disc breakdown requiring revision surgeries at the cranial or caudal adjacent segments. Additionally, the sagittal alignment parameters, including SVA, CL, T1S, and CL correction, were not significantly different between the hardware failure group and the no hardware failure group (Table 2).

**Table 2:** Changes of radiological parameters between preoperative period and final follow-up

	Hardware failure	No hardware failure	*p-*Value
C2-C7 CL (degree)	Mean **±** SD		
Pre-operative	13.5 ± 6.9	14.7 ± 6.5	0.545
Final follow-up	17.8 ± 6.2	18.5 ± 6.5	0.701
C2-C7 SVA (mm)			
Pre-operative	23.8 ± 10.5	22.5 ± 8.9	0.641
Final follow-up	21.4 ± 14.3	19.8 ± 15.3	0.567
T1S (degree)			
Pre-operative	23.8 ± 9.2	26.1 ± 9.0	0.436
Final follow-up	22.6 ± 13.6	24.5 ± 10.5	0.502
CL correction (degree)	4.3 ± 1.1	3.8 ± 1.2	0.196

CL, cervical lordosis, SVA, sagittal vertical axis; T1S, T1 Slope; SD, standard deviation

### Regional hounsfield units

Considering the difference in screw trajectory between the PS and LMS, we measured their regional HUs separately. The regional HU of PS and LMS in the hardware failure group was lower than that in the no hardware failure group (PS: 267 ± 45 vs. 368 ± 43, p = 0.001; LMS: 308 ± 53 vs. 412 ± 41, p = 0.001) (Table 1).

### Other Complications

No major neurological or wound complications were observed in either group, and there was no revision surgery performed in either group.

## Discussion

The hardware failure rate after laminectomy and PCF in our study was 25%, which is aligned with and add to prior literature [6, 7]. We found osteoporosis, fixation ended at C7, and low regional HU of screw trajectory were the independent risk factors. The novelty of the current study is that we used regional HU of screw trajectory as an evaluation index, instead of using the HU of entire vertebral body.

Bone fusion is by far the most important factor in preventing hardware failure. The fusion technique, the patient’s medical condition and activity, and the gap to be fused all play a role in mechanical failure rates [7, 8]. In cervical spinal surgery, due to the surgical operation space being comparatively limited and obstructed by the implants, the area and volume of the bone graft are very small [8]. Previous studies on the risk factors for PCF, such as RA, tumour, infection and trauma, did not disregard the potential impact of bone fusion nor the potential impact of the absence of measures on fusion promotion [6, 7]. To mitigate the impact of the aforementioned factors on the outcomes, all patients in this study underwent identical surgical procedures under the guidance of the same team, and any potential factors that may impact bone fusion, including RA, tumour, infection, trauma, and so forth were disregarded. In addition, small wedges of autografts from the lamina were placed adjacent to the bilateral joints to facilitate fusion. Previous studies have suggested that the bone fusion rate following posterior cervical fusion decreases with the number of fusion levels and has been reported to range from 70 to 95% [8]. Following the implementation of the aforementioned strategies in our study, the fusion rate of both groups exceeded 90%.

Vertebral bone quality plays a significant role in determining fixation. According to previous studies, bone mineral density (BMD) has a positive impact on ultimate compressive strength, and there is a linear increasing relationship between stress and BMD [9, 10]. Yamagata et al. [11] reported that a 100 mg/cm^2^ decrease in the BMD caused a 10 kP decrease in the pullout strength. Other researchers also reported a strong correlation between the pullout strength of screws and BMD [12]. In terms of BMD assessment, dual-energy X-ray absorptiometry (DEXA) is considered the “gold standard” due to its simplicity and cost-effectiveness, with low-level radiation exposure. In this study, we diagnosed osteoporosis with a DEXA result (T score) less than 2.5. The hardware failure rate of patients with osteoporosis was significantly lower than that of patients without osteoporosis (57.1% vs. 14.3%), which was consistent with prior studies.

Although DEXA is clearly effective, there are several methodological constraints for quantifying BMD in patients with a degenerative spine. The existence of osteophytes, articular hypertrophy, and soft-tissue deterioration, such as abdominal vascular wall calcification, would influence the lumbar BMD value and lead to its overestimation [13]. Recently, BMD assessment using HU has been developed as a new trustworthy approach to measure bone quality. HU values have been found to be favourably linked with both vertebral compressive strength and DEXA-measured BMD [14]. Following spinal fusion, decreased HU values of the vertebral body were related to nonunion, interbody cage subsidence, and adjacent segment fractures [15, 16]. In our study, we conducted a separate recording and comparison of regional HU of screw trajectory for loosened/broken and fixed PS/LMS screws, taking into account the disparity in screw trajectory between the two. The regional HU of the screw trajectory of the loosened/broken screw was significantly lower than that of the fixed screw (PS: 267 vs. 368, LMS: 308 vs. 412). Few studies have reported the HU of specific regions of the vertebra as an objective index for screw fixation in the field of spinal fixation surgery. In a study of 92 patients who underwent single-level posterior lumbar interbody fusion, Matsukawa et al. [17] found that the regional HU value of the screw trajectory (r = 0.75) had a stronger correlation with insertional torque than femoral BMD (r = 0.59) and lumbar BMD (r = 0.55) and that it was an independent risk. It would be useful for predicting screw stability before surgery. Surgeons can choose a preferred screw size and screw trajectory in advance using preoperative CT modelling of screw placement to achieve optimal fixation [18]. In lumbar fixation surgery, surgeons can make adjustments to improve screw purchase before insertion, such as adjusting the screw size, inserting cement or hydroxyapatite-stick into the screw hole, and using expandable screws if the regional HU values of the screw trajectory do not meet a certain threshold. These benefits may significantly contribute to the improvement of the bone-screw interface integrity, resulting in a lower risk of screw loosening and more effective fusion. However, the above measures are less commonly used in cervical fixation surgery, owing to the lack of related products and the increased hazard of bone cement leakage to the cervical spinal canal [19, 20].

The authors also found that the fixation endpoint was a significant risk factor of hardware failure. Patients with fixation endpoints that did not cross the cervicothoracic junction (CTJ) had a higher risk of hardware failure after laminectomy and PCF. The CTJ has unique biomechanical functions, as the relatively rigid thoracic spine transitions at this locus into the relatively flexible cervical spine [21, 22]. Previous studies have identified the CTJ as a site at risk of postoperative complications due to its inherent structural vulnerability [23]. In addition, instrumentation terminating at the CTJ provides a larger moment arm at this already stressed segment [24], and posterior approaches involving the cervical spine are more disruptive of posterior tension band structures and cause further instability of the CTJ [25]. The results of our study were consistent with previous reports. Ibaseta et al. [26] concluded that crossing the CTJ during cervical arthrodesis does not increase operative risk and lowers revision rates by reducing the risk of adjacent segment disease (ASD). Schroeder et al. [27] also concluded that multilevel posterior cervical fusion should be extended to T1 because not crossing the CTJ increases ASD risk and reoperation rates at the C7-T1 junction.

Numerous studies have elucidated the link between sagittal alignment and health-related quality of life (HRQOL) outcomes [28–30]. Patients with poor sagittal alignment have increased energy expenditure during activities and at rest, and they often develop painful compensatory alignment changes to maintain upright posture, including knee flexion, pelvic retroversion, thoracic hypokyphosis, and cervical hyperlordosis [28]. However, most of the current studies have focused on the relationship between sagittal alignment and quality of life, and few have investigated the relationship between sagittal alignment and hardware failure. The compensatory changes brought about by the sagittal alignment can also exert force on the cervical internal fixation, resulting in hardware failure. Unfortunately, no relationship between sagittal alignment parameters (SVA, T1S, CL, and CL correction) and failure was found in our study. On the one hand, this may be attributed to the fact that most of our patients were without cervical kyphosis, which meant that very few corrective procedures were performed in our series. Prior studies [28, 31] have shown that the relationships between sagittal alignment and HRQOL were not significant in patients with radiculopathy but appeared to be particularly pronounced in patients with cervical deformity, and the compensatory changes brought about by sagittal alignment may not be enough to cause hardware failure.

The screw density was similar in the hardware failure and no hardware failure groups (1.5 ± 0.2 vs. 1.7 ± 0.3) in our series. Our findings align with and add to prior literature, which has shown that high screw density brings high stiffness [32] to facilitate immediate stability after surgery. Some researchers have recommended that more fixators be applied at more levels during surgeries that severely destabilize the stability of the spine [33]. However, changes in alignment in fusion levels and high stiffness may lead to hardware failure [32, 34].

All implant failures were asymptomatic, and no major neurologic or wound complications or revision surgery were observed in either group. This was consistent with the report by Deen et al. [35], who analysed complications incurred by 100 patients treated with the cervical lateral mass screw-rod system. They reported two cases of screw breakage, both of which were asymptomatic. This may be attributed to the mechanical loading in the cervical spine being far less than that in the lumbar spine [36], and the residual implants maintain enough stability to allow bone fusion without de novo symptoms. Among our 14 hardware failure cases, only one occurred at C6, and the others occurred at the upper or lower end levels (C2 or C3, C7 or T1). In multilevel fusion, the most critical site to be fused is generally located in the middle of the construct. One reason for the lack of de novo symptoms could be that the implant remained stabilized at the critical site, despite the failure at the end of the construct [7].

### Limitations

There were some limitations in our study. Our study was a single-centre retrospective study, the sample size of this study was relatively small, the follow-up period was short, and no complications, such as ASD requiring treatment, were found. In our study, both LMS and PS were implanted in all patients, which led to inconsistencies in screw placement and biomechanical strength. Their impact on hardware failure was not analysed. In addition, when exploring whether osteoporosis is a risk factor of hardware failure after PCF, patients with lower instrumented end vertebra at C7 and T1 should be categorized into groups with and without hardware failure, respectively. Unfortunately, the number of patients in this retrospective study is too small for the above statistical analysis. Finally, the length and diameter of the screws were not considered. In conclusion, further studies are needed to avoid selection bias, and long-term prospective or randomized control trials investigating the risk factors of hardware failure after long-segment PCF are necessary to provide optimal clinical evidence.

## Conclusions

Osteoporosis, fixation ended at C7, and low regional HU of screw trajectory were the independent risk factors of hardware failure after laminectomy and PCF. Future studies should illuminate if preventive measures targeting these factors can help reduce hardware failure and identified more risk factors, and perform long-term follow-up.

### Electronic supplementary material

Below is the link to the electronic supplementary material.

**Supplementary Material 1**: (STROBE-checklist)

## Data Availability

The results/data/figures in this manuscript have not been published elsewhere, nor are they under consideration by another publisher. The datasets used and/or analysed during the current study are available from the corresponding author upon reasonable request.
