# Supplementary material for: Risk factors of instrumentation failure after laminectomy and posterior cervical fusions (PCF)
Source: BMC Musculoskelet Disord. 2024 Jan 2;25:1. doi: 10.1186/s12891-023-07116-z (PMC10759594; doi:10.1186/s12891-023-07116-z)
Supplement: Supplementary file 1 — Supplementary Material 1: (STROBE-checklist) [file 12891_2023_7116_MOESM1_ESM.docx]

STROBE Statement—checklist of items that should be included in reports of observational studies

|  | Item No. | Recommendation | Page  No. | Relevant text from manuscript |
| --- | --- | --- | --- | --- |
| **Title and abstract** | 1 | (*a*) Indicate the study’s design with a commonly used term in the title or the abstract | 1 | The present study was a retrospective, single centre, observational study. |
|  |  | (*b*) Provide in the abstract an informative and balanced summary of what was done and what was found | 1,2 | Patients who underwent laminectomy and PCF with instrumentation in a single institution between January 2019 and January 2021 were included. Patients were divided into hardware failure and no hardware failure group according to whether there was a hardware failure. Data, including sex, age, screw density, end vertebra (C7 or T1), cervical sagittal alignment parameters (C2-C7 cervical lordosis, C2-C7 sagittal vertical axis, T1 slope, Cervical lordosis correction), regional Hounsfield units (HU) of the screw trajectory and osteoporosis status, were collected and compared between the two groups.Osteoporosis, fixation ending at C7, and low regional HU value of the screw trajectory were the independent risk factors of hardware failure after laminectomy and PCF. Future studies should illuminate if preventive measures targeting these factors can help reduce hardware failure and identified more risk factors, and perform long-term follow-up. |
| Introduction | | | |  |
| Background/rationale | 2 | Explain the scientific background and rationale for the investigation being reported | 2 | Due to the rapid changes in modern production and lifestyle, the prevalence of cervical myelopathy is 3.8% to 17.6% [1]. Though the prevalence in various regions vary, the number of patients increases by year [1]. PCF with instrumentation is performed to treat degenerative diseases such as cervical myelopathy, ossification of the posterior longitudinal ligament (OPLL), and multilevel cervical radiculopathy [2]. Decompression relieves pressure on the spinal cord, and fixation helps correct and maintain cervical alignment and stability. The annual rate of PCF in adults in the United States with a preoperative diagnosis of degenerative disease increased 2.7-fold from 2001 to 2013, and the annual number of PCF conducted on individuals with a preoperative diagnosis of cervical spondylotic myelopathy (CSM) increased 2.9-fold from 2003 to 2013 [2]. Although there are various types of screws and techniques for screw insertion in the cervical spine, hardware failure is one of the most common complications [3]-[8]. Hardware failure is defined as screw or rod breakage, screw loosening, or nonunion. The failure rates ranged from 6.1 to 38.9% and may even exceed 50% after PCF [6][7]. The incidence of hardware failure leading to surgical revision ranged from 16.7% to 42.8%, with a pooled incidence of 22.7% [2]-[6]. In addition, hardware failure may also cause pseudarthrosis, chronic pain, and neurologic deficits [2][3][6]. However, there have been few reports focusing on the characteristics and risk factors of hardware failure in laminectomy and PCF[3][4][5]. To assess this common postoperative complication, a thorough understanding of the characteristics and risk factors of hardware failure after laminectomy and PCF is needed. |
| Objectives | 3 | State specific objectives, including any prespecified hypotheses | 2 | Therefore, we conducted the present study to elucidate the characteristics and risk factors of hardware failure in laminectomy and PCF. |
| Methods | | | |  |
| Study design | 4 | Present key elements of study design early in the paper | 2 | The present study was a retrospective, single centre, observational study. (line 42) |
| Setting | 5 | Describe the setting, locations, and relevant dates, including periods of recruitment, exposure, follow-up, and data collection | 2,3 | Data of patients following laminectomy and PCF with instrumentation between January 2019 and January 2021, including sex, age, screw density, end vertebra (C7 or T1), cervical sagittal alignment parameters (C2-C7 cervical lordosis (CL), C2-C7 sagittal vertical axis, T1 slope, CL correction), regional Hounsfield units (HU) of screw trajectory, and osteoporosis status were collected, which aimed to investigate risk factors of hardware failure after laminectomy and PCF with instrumentation. This study was approved by The Ethics Committee of The Second Xiangya Hospital of Central South University (NO.20191243) in January 2019. (page 2, line 42-44; page 3, line 1-5) |
| Participants | 6 | (*a*) *Cohort study*—Give the eligibility criteria, and the sources and methods of selection of participants. Describe methods of follow-up  *Case-control study*—Give the eligibility criteria, and the sources and methods of case ascertainment and control selection. Give the rationale for the choice of cases and controls  *Cross-sectional study*—Give the eligibility criteria, and the sources and methods of selection of participants | 3 | The inclusion criteria were as follows: (1) patients who underwent 4-level and above laminectomy and PCF. The exclusion criteria were as follows: (1) follow-up less than 1 year; (2) age less than 18 years; and (3) cervical spinal surgery for infection, trauma, malignancy, or rheumatoid arthritis (RA). (line 10-13) |
|  |  | (*b*) *Cohort study*—For matched studies, give matching criteria and number of exposed and unexposed  *Case-control study*—For matched studies, give matching criteria and the number of controls per case | 3 | Patients were divided into hardware failure (n = 14) and no hardware failure group (n = 42) according to whether there was a hardware failure. |
| Variables | 7 | Clearly define all outcomes, exposures, predictors, potential confounders, and effect modifiers. Give diagnostic criteria, if applicable | 2,3 | Data of patients following laminectomy and PCF with instrumentation between January 2019 and January 2021, including sex, age, screw density, end vertebra (C7 or T1), cervical sagittal alignment parameters (C2-C7 cervical lordosis (CL), C2-C7 sagittal vertical axis, T1 slope, CL correction), regional Hounsfield units (HU) of screw trajectory, and osteoporosis status were collected. |
| Data sources/ measurement | 8* | For each variable of interest, give sources of data and details of methods of assessment (measurement). Describe comparability of assessment methods if there is more than one group | 3,4 | The fixation level, screw density (total number of screws/actual fixation level), regional HU of the PS/LMS screw trajectory, and DXA results (T score) were recorded. Osteoporosis was diagnosed by DXA (T score less than -2.5). T score is to compare the bone quality of the subject with that of a normal young man of the same sex to determine whether there is osteoporosis (Normal: -1 to +1; Low bone mass: -1 to -2.5; Osteoporosis: less than -2.5) [12]. Radiological examination was used to assess hardware failure. Osseointegrated screws do not show any sign of radiolucency around their edges in planar radiographs. Screw loosening was detected by the presence of a radiolucent area greater than 1 mm or the presence of a “double halo,” which is defined as an inner radiolucent zone surrounded by an outer radiopaque rim of dense bone [8]. Screw nut loosening occurred when the nut became dislodged from the screw head and could be seen as a gap between the screw grooves and the ridge [7]. Screw or rod breakage can be seen with obvious cracks and/or angulation in anteroposterior or lateral radiographs [8]. Nonunion was defined as a lack of bridging osseous trabeculae between the involved vertebrae [7], >2 mm motion between the affected spinous processes on flexion-extension lateral radiographs, or >2° of motion on flexion-extension radiographs at the 12-month follow-up [8]. When needed, computed tomography (CT) images were obtained to confirm the presence of nonunion. For HU measurement, all patients were assessed by a helical 64-channel CT scanner (Aquilion 64®, Toshiba Medical, Otawara, Japan). The position of each screw was extracted from postoperative CT images obtained immediately after surgery and superimposed three-dimensionally on the vertebra of the preoperative CT by referring to the vertebral anatomical landmarks (Fig. 2) [17]. The cylindrical area along each screw with an outer diameter was placed on the vertebra, and density information was collected for all voxels contacting the sample. Average HU values were calculated automatically from the entry point at the lamina to the screw tip per 1 mm section orthogonal to the screw axis.  To evaluate the sagittal alignment, we also measured the following parameters through the cervical spine (standing position) radiograph preoperatively and at the last follow-up: (1) C2-C7 sagittal vertical axis (C2-C7 SVA); (2) T1 slope (T1S); (3) C2-C7 Cervical lordosis (CL); (4) CL correction (postoperative C2-C7 CL minus preoperative C2-C7 CL) (Fig. 3). |
| Bias | 9 | Describe any efforts to address potential sources of bias | 4 | All patient imaging data (numbered but no patient information) were distributed to two spine surgeons (Dr. Pan and Dr. Yuan), who judged the occurrence of hardware failure based on the previous criteria, and if they agreed, no one else judged again. If there was a disagreement, a senior spine surgeon (Dr. Lv) was invited to participate in the judgement and make ultimate judgement.  All measurements were performed by two independent researchers (Dr. Pan and Dr. Yuan), and the results of their measurements were analysed by the intraclass correlation coefficient for data consistency. Disagreements were discussed with another independent expert (Dr. Lv), and a consensus was reached to minimize observer bias. |
| Study size | 10 | Explain how the study size was arrived at | 3 | Patients were divided into hardware failure (n = 14) and no hardware failure group (n = 42) according to whether there was a hardware failure. |

Continued on next page

| Quantitative variables | 11 | Explain how quantitative variables were handled in the analyses. If applicable, describe which groupings were chosen and why | 4 | The comparison between two groups was performed via Student’s t test, chi-square test and paired t tests in Statistical Product and Service Solutions 28.0 statistical software (SPSS, Inc., Chicago, Illinois, USA). |
| --- | --- | --- | --- | --- |
| Statistical methods | 12 | (*a*) Describe all statistical methods, including those used to control for confounding | 4 | The comparison between two groups was performed via Student’s t test, chi-square test and paired t tests in Statistical Product and Service Solutions 28.0 statistical software (SPSS, Inc., Chicago, Illinois, USA). |
|  |  | (*b*) Describe any methods used to examine subgroups and interactions | 4 | The comparison between two groups was performed via Student’s t test, chi-square test and paired t tests in Statistical Product and Service Solutions 28.0 statistical software (SPSS, Inc., Chicago, Illinois, USA). |
|  |  | (*c*) Explain how missing data were addressed |  | no |
|  |  | (*d*) *Cohort study*—If applicable, explain how loss to follow-up was addressed  *Case-control study*—If applicable, explain how matching of cases and controls was addressed  *Cross-sectional study*—If applicable, describe analytical methods taking account of sampling strategy |  | no |
|  |  | (*e*) Describe any sensitivity analyses |  | no |
| Results | | | | |
| Participants | 13* | (a) Report numbers of individuals at each stage of study—eg numbers potentially eligible, examined for eligibility, confirmed eligible, included in the study, completing follow-up, and analysed | 4 | We analysed the clinical data of 56 patients in total. The mean follow-up time was 20.6 months (range 12-30 months), and the average age of the patients was 55.6 years (range 36-81 years). Patients were divided into the hardware failure group (n = 14) and no hardware failure group (n = 42). |
|  |  | (b) Give reasons for non-participation at each stage |  | Fig.1 |
|  |  | (c) Consider use of a flow diagram |  | Fig.1 |
| Descriptive data | 14* | (a) Give characteristics of study participants (eg demographic, clinical, social) and information on exposures and potential confounders | 4 | There were no significant differences in the general information (age, sex, follow-up period) of the patients between the two groups. |
|  |  | (b) Indicate number of participants with missing data for each variable of interest |  | no |
|  |  | (c) *Cohort study*—Summarise follow-up time (eg, average and total amount) | 4 | We analysed the clinical data of 56 patients in total. The mean follow-up time was 20.6 months (range 12-30 months), and the average age of the patients was 55.6 years (range 36-81 years). |
| Outcome data | 15* | *Cohort study*—Report numbers of outcome events or summary measures over time |  |  |
|  |  | *Case-control study—*Report numbers in each exposure category, or summary measures of exposure | 5 | The differences in fusion rate, fixation level, and screw density between the two groups were not statistically significant (p>0.05). We analysed a total of 479 screws (PS: 161, LMS:318), 30 of which had problems including screw loosening, breakage, or back out (PS:17, LMS:13). There was no loosening, breakage of the screw nut or rod breakage. The failure rate of the lower fixation endpoint at T1 was lower than that at C7 (9% vs. 36.3%, p = 0.019). The hardware failure rate in patients without osteoporosis was lower than that in patients with osteoporosis (14.3% vs. 57.1%, p = 0.001) (Table 1). No patient in either group had any obvious instability or disc breakdown requiring revision surgeries at the cranial or caudal adjacent segments. Additionally, the sagittal alignment parameters, including SVA, CL, T1S, and CL correction, were not significantly different between the hardware failure group and the no hardware failure group (Table 2). Considering the difference in screw trajectory between the PS and LMS, we measured their regional HUs separately. The regional HU of PS and LMS in the hardware failure group was lower than that in the no hardware failure group (PS: 267±45 vs. 368±43, p = 0.001; LMS: 308±53 vs. 412±41, p = 0.001) (Table 1). |
|  |  | *Cross-sectional study—*Report numbers of outcome events or summary measures |  |  |
| Main results | 16 | (*a*) Give unadjusted estimates and, if applicable, confounder-adjusted estimates and their precision (eg, 95% confidence interval). Make clear which confounders were adjusted for and why they were included | 5 | The differences in fusion rate, fixation level, and screw density between the two groups were not statistically significant (p>0.05). We analysed a total of 479 screws (PS: 161, LMS:318), 30 of which had problems including screw loosening, breakage, or back out (PS:17, LMS:13). There was no loosening, breakage of the screw nut or rod breakage. The failure rate of the lower fixation endpoint at T1 was lower than that at C7 (9% vs. 36.3%, p = 0.019). The hardware failure rate in patients without osteoporosis was lower than that in patients with osteoporosis (14.3% vs. 57.1%, p = 0.001) (Table 1). No patient in either group had any obvious instability or disc breakdown requiring revision surgeries at the cranial or caudal adjacent segments. Additionally, the sagittal alignment parameters, including SVA, CL, T1S, and CL correction, were not significantly different between the hardware failure group and the no hardware failure group (Table 2). Considering the difference in screw trajectory between the PS and LMS, we measured their regional HUs separately. The regional HU of PS and LMS in the hardware failure group was lower than that in the no hardware failure group (PS: 267±45 vs. 368±43, p = 0.001; LMS: 308±53 vs. 412±41, p = 0.001) (Table 1). |
|  |  | (*b*) Report category boundaries when continuous variables were categorized | 5 | The differences in fusion rate, fixation level, and screw density between the two groups were not statistically significant (p>0.05). We analysed a total of 479 screws (PS: 161, LMS:318), 30 of which had problems including screw loosening, breakage, or back out (PS:17, LMS:13). There was no loosening, breakage of the screw nut or rod breakage. The failure rate of the lower fixation endpoint at T1 was lower than that at C7 (9% vs. 36.3%, p = 0.019). The hardware failure rate in patients without osteoporosis was lower than that in patients with osteoporosis (14.3% vs. 57.1%, p = 0.001) (Table 1). No patient in either group had any obvious instability or disc breakdown requiring revision surgeries at the cranial or caudal adjacent segments. Additionally, the sagittal alignment parameters, including SVA, CL, T1S, and CL correction, were not significantly different between the hardware failure group and the no hardware failure group (Table 2). Considering the difference in screw trajectory between the PS and LMS, we measured their regional HUs separately. The regional HU of PS and LMS in the hardware failure group was lower than that in the no hardware failure group (PS: 267±45 vs. 368±43, p = 0.001; LMS: 308±53 vs. 412±41, p = 0.001) (Table 1). |
|  |  | (*c*) If relevant, consider translating estimates of relative risk into absolute risk for a meaningful time period | 5 | The differences in fusion rate, fixation level, and screw density between the two groups were not statistically significant (p>0.05). We analysed a total of 479 screws (PS: 161, LMS:318), 30 of which had problems including screw loosening, breakage, or back out (PS:17, LMS:13). There was no loosening, breakage of the screw nut or rod breakage. The failure rate of the lower fixation endpoint at T1 was lower than that at C7 (9% vs. 36.3%, p = 0.019). The hardware failure rate in patients without osteoporosis was lower than that in patients with osteoporosis (14.3% vs. 57.1%, p = 0.001) (Table 1). No patient in either group had any obvious instability or disc breakdown requiring revision surgeries at the cranial or caudal adjacent segments. Additionally, the sagittal alignment parameters, including SVA, CL, T1S, and CL correction, were not significantly different between the hardware failure group and the no hardware failure group (Table 2). Considering the difference in screw trajectory between the PS and LMS, we measured their regional HUs separately. The regional HU of PS and LMS in the hardware failure group was lower than that in the no hardware failure group (PS: 267±45 vs. 368±43, p = 0.001; LMS: 308±53 vs. 412±41, p = 0.001) (Table 1). |

Continued on next page

| Other analyses | 17 | Report other analyses done—eg analyses of subgroups and interactions, and sensitivity analyses |  | no |
| --- | --- | --- | --- | --- |
| Discussion | | | | |
| Key results | 18 | Summarise key results with reference to study objectives | 5 | The hardware failure rate after laminectomy and PCF in our study was 25%, which is aligned with and add to prior literature [6][7]. We found osteoporosis, fixation ended at C7, and low regional HU of screw trajectory were the independent risk factors. The novelty of the current study is that we used regional HU of screw trajectory as an evaluation index, instead of using the HU of entire vertebral body. |
| Limitations | 19 | Discuss limitations of the study, taking into account sources of potential bias or imprecision. Discuss both direction and magnitude of any potential bias | 7,8 | There were some limitations in our study. Our study was a single-centre retrospective study, the sample size of this study was relatively small, the follow-up period was short, and no complications, such as ASD requiring treatment, were found. In our study, both LMS and PS were implanted in all patients, which led to inconsistencies in screw placement and biomechanical strength. Their impact on hardware failure was not analysed. In addition, when exploring whether osteoporosis is a risk factor of hardware failure after PCF, patients with lower instrumented end vertebra at C7 and T1 should be categorized into groups with and without hardware failure, respectively. Unfortunately, the number of patients in this retrospective study is too small for the above statistical analysis. Finally, the length and diameter of the screws were not considered. |
| Interpretation | 20 | Give a cautious overall interpretation of results considering objectives, limitations, multiplicity of analyses, results from similar studies, and other relevant evidence | 8 | Osteoporosis, fixation ended at C7, and low regional HU of screw trajectory were the independent risk factors of hardware failure after laminectomy and PCF. Future studies should illuminate if preventive measures targeting these factors can help reduce hardware failure and identified more risk factors, and perform long-term follow-up. |
| Generalisability | 21 | Discuss the generalisability (external validity) of the study results | 8 | In conclusion, further studies are needed to avoid selection bias, and long-term prospective or randomized control trials investigating the risk factors of hardware failure after long-segment PCF are necessary to provide optimal clinical evidence. |
| Other information | |  | | |
| Funding | 22 | Give the source of funding and the role of the funders for the present study and, if applicable, for the original study on which the present article is based | 9 | No funding |

*Give information separately for cases and controls in case-control studies and, if applicable, for exposed and unexposed groups in cohort and cross-sectional studies.

**Note:** An Explanation and Elaboration article discusses each checklist item and gives methodological background and published examples of transparent reporting. The STROBE checklist is best used in conjunction with this article (freely available on the Web sites of PLoS Medicine at http://www.plosmedicine.org/, Annals of Internal Medicine at http://www.annals.org/, and Epidemiology at http://www.epidem.com/). Information on the STROBE Initiative is available at www.strobe-statement.org.
